# Markers Specific to *Bacteroides fragilis* Group Bacteria as Indicators of Anthropogenic Pollution of Surface Waters

**DOI:** 10.3390/ijerph17197137

**Published:** 2020-09-29

**Authors:** Sebastian Niestępski, Monika Harnisz, Ewa Korzeniewska, Adriana Osińska

**Affiliations:** Department of Engineering of Water Protection and Environmental Microbiology, Faculty of Geoengineering, University of Warmia and Mazury in Olsztyn, Prawocheńskiego 1, 10-720 Olsztyn, Poland; sebastian.niestepski@uwm.edu.pl (S.N.); ewa.korzeniewska@uwm.edu.pl (E.K.); adriana.osinska@uwm.edu.pl (A.O.)

**Keywords:** antibiotics resistance genes, anthropogenic pollution, *Bacteroides fragilis* group bacteria

## Abstract

The aim of this study was to evaluate the applicability of markers specific to *Bacteroides fragilis* group (BFG) bacteria as indicators of anthropogenic pollution of surface waters. In addition, the impact of wastewater treatment plants (WWTPs) on the spread of genes specific to fecal indicator bacteria and genes encoding antimicrobial resistance in water bodies was also determined. Samples of hospital wastewater (HWW), untreated wastewater (UWW), and treated wastewater (TWW) evacuated from a WWTP were collected, and samples of river water were taken upstream (URW) and downstream (DRW) from the wastewater discharge point to determine, by qPCR, the presence of genes specific to BFG, *Escherichia coli* and *Enterococcus faecalis*, and the abundance of 11 antibiotic resistance genes (ARGs) and two integrase genes. The total number of bacterial cells (TCN) in the examined samples was determined by fluorescence in situ hybridization (FISH). Genes specific to BFG predominated among the analyzed indicator microorganisms in HWW, and their copy numbers were similar to those of genes specific to *E. coli* and *E. faecalis* in the remaining samples. The abundance of genes specific to BFG was highly correlated with the abundance of genes characteristic of *E. coli* and *E. faecalis*, all analyzed ARGs and *int*I genes. The results of this study indicate that genes specific to BFG can be used in analyses of human fecal pollution, and as indicators of environmental contamination with ARGs. A significant increase in the copy numbers of genes specific to BFG, *E. coli*, and seven out of the 11 analyzed ARGs was noted in samples of river water collected downstream from the wastewater discharge point, which suggests that WWTPs are an important source of these genes in riparian environments.

## 1. Introduction

The microbiological quality of surface waters has to be monitored to ensure their sanitary safety. According to European Union standards, the sanitary quality of surface water is evaluated based mainly on the enumeration of *Escherichia coli*, coliform bacteria, and intestinal enterococci in water samples. The presence of these bacteria in water samples points to recent contamination of aquatic environments with fecal matter [1,2,3,4]. These analyses rely on culture-based laboratory techniques (such as, e.g., the most-probable-number and membrane filtration methods), which are cheap and simple to perform, but do not clearly identify the source of contamination. *Escherichia coli* and *Enterococcus* bacteria are present in both human and animal feces; therefore, the source of pollution cannot be accurately determined [5,6]. Moreover, a growing body of research suggests that *E. coli* and *Enterococcus* indicator bacteria originate not only from human and animal feces, but also from contaminated soil, sewage sludge, or even algae farms [7,8]. Additional indicators, e.g., based on specific markers of human fecal pollution, are needed to expand the range of the existing standard methods and overcome their limitations in monitoring the microbiological quality of surface waters [9,10,11,12]. The application of specific indicators of human fecal pollution would enhance the sensitivity of microbiological quality assessments and enable precise identification of the sources of environmental contamination.

Bacteria of the family *Bacteroides* predominate in the human gut microbiota [13,14], therefore they could be used as potential indicators of water contamination with human feces. Analyses of genetic markers specific to Bacteroides bacteria colonizing the human gut, based on PCR and qPCR assays, have become popular tools for tracking the sources of microbial contamination in surface waters in recent years [10,15,16,17]. The application of markers specific to human-associated *Bacteroides* sp. would support the unambiguous identification of water pollution sources, such as household wastewater or treated sewage.

Antibiotic resistance constitutes a global health problem [18]. The widespread use of antibiotics in human and veterinary medicine has accelerated the spread of antibiotic resistance determinants in the environment [19]. The presence of antibiotic-resistant bacteria (ARB) and antibiotic resistance genes (ARGs) in the natural environment is often associated with human activities, such as aquaculture, livestock farming, and evacuation of treated municipal wastewater to surface water bodies [20]. Fecal *E. coli*, coliforms, and enterococci are the most frequently analyzed bacteria that are isolated from treated wastewater [21,22]. Bacterial strains resistant to various groups of antibiotics are widely identified. Antibiotic resistance genes are localized on mobile genetic elements, such as plasmids, transposons, and integrons, which facilitates the spread of antibiotic resistance between bacteria of the same and different origin via horizontal gene transfer (HGT) [23]. Research has demonstrated that ARG-harboring plasmids are transferred between various strains of *E. faecalis* and between *E. faecalis* and *E. coli* in wastewater [21]. Niestępski et al. [13,24] have recently reported high levels of antibiotic resistance and considerable diversity of ARGs in Bacteroides fragilis group (BFG) strains isolated from hospital wastewater and wastewater treatment plants (WWTPs), as well as the widespread coexistence of genes specific to BFG and resistance genes in wastewater and rivers receiving treated sewage. These observations suggest that fecal indicator bacteria could be robust indicators of water contamination with ARGs.

Microbial counts in wastewater are reduced 10- to 100-fold during treatment [25]. Despite the above, considerable amounts of ARB and ARGs are still present in treated wastewater which is evacuated to surface water bodies and reaches ground water [20,26,27,28,29,30]. Korzeniewska and Harnisz [28], Czekalski et al. [31], and Zhang et al. [32] demonstrated that total bacterial counts are reduced during specific wastewater treatment processes, such as disinfection, but the percentage of ARB and, consequently, ARGs in the bacterial community can increase. Previous studies have shown that WWTPs can be sources of drug-resistant and multidrug-resistant bacteria, such as *E. coli* and *Bacteroides* sp., in surface waters [24,33,34].

The potential spread of environmental ARB and ARGs and the transfer of ARGs from environmental bacteria to human pathogens compromise the effectiveness of antimicrobial drugs, which can have serious implications for public health [35]. The markers associated with HGT, such as integrons, are often identified in locations that are subjected to high levels of anthropogenic pressure, including in environments contaminated with wastewater [36]. Recent research has confirmed that ARGs and integrase genes are effective indicators of human-caused pollution [37,38,39].

The above observations suggest that a new indicator which supports simultaneous assessments of fecal contamination, as well as contamination with ARB and/or ARGs, should be introduced to water quality analyses. Therefore, the aim of this study was to evaluate the applicability of markers specific to BFG bacteria in analyses of the microbiological quality of surface waters. The following research hypotheses were formulated and tested: (i) markers specific to BFG bacteria can be used as indicators of anthropogenic pollution of surface waters; (ii) wastewater treatment plants are sources of dissemination of genes specific to fecal indicator bacteria and genes encoding antimicrobial resistance in the environment.

## 2. Materials and Methods

### 2.1. Sample Collection

Samples of hospital wastewater (HWW, 100 mL), untreated wastewater (UWW, 100 mL), and treated wastewater evacuated from the Łyna Wastewater Treatment Plant in Olsztyn, Poland (TWW, 300 mL), as well as samples of river water collected from the Łyna River around 600 m upstream and downstream from the wastewater discharge point (URW and DRW, 500 mL each), were analyzed in this study. The samples were collected into sterile bottles in winter (February) and summer (June) of 2019, and they were transported at a temperature of 4 °C to the laboratory for further analyses. Samples of UWW were collected at the outlet of the coarse screen chamber.

### 2.2. Isolation of Genomic DNA from Wastewater and River Water Samples

All wastewater and river water samples were passed through standard polycarbonate membrane filters with a hydrophobic edge (0.2 μm pore size) (Merck, Millipore, Burlington, MA, USA). Filters containing sludge were cut into small pieces and transferred to sterile test tubes (2 mL). Tube contents were combined with 1.5 mL of 1 × PBS, and the tubes were shaken at 200 rpm for 3 h at room temperature. Genomic DNA was extracted with the Fast DNA SPIN Kit for Soil (MP Biomedicals, Irvine, CA, USA) according to the manufacturer’s instructions. The concentration and quality of the isolated DNA were determined with the Nanodrop spectrophotometer (NanoDrop^®^ ND-1000, NanoDrop Technologies, Wilmington, DE, USA). Genomic DNA was stored at a temperature of −20 °C until analysis.

### 2.3. Determination of Total Number of Bacterial Cells by Fluorescence In Situ Hybridization (FISH)

The total number of bacterial cells (TCN) was determined by FISH and DAPI methods in 10 mL specimens obtained from each sample of HWW, UWW, and TWW, and in 40 mL specimens obtained from each sample of river water (URW and DRW). The specimens were fixed in freshly prepared paraformaldehyde solution (pH 7.4, final concentration of 4%) and stored at room temperature for 1 h. A set of serial solutions was made, and the fixed samples were passed through white polycarbonate filters (0.2 µm pore size) (Merck, Millipore, Burlington, MA, USA) under low negative pressure. The filters were twice rinsed with 20 mL of ultrapure water (dddH_2_O), dried at room temperature, and stored on Petri plates at a temperature of −20 °C until analysis.

The TCN was determined under an epifluorescence microscope (BX61, Olympus, Tokyo, Japan) by analyzing filter fragments stained with 4′,6-diamidino-2-phenylindole (DAPI, final concentration of 0.1 μg/mL), with the use of a 16S rRNA-targeted EUB338 probe (hybridized to position 338–355 bp) labeled with Cy3 cyanine dye. All samples were simultaneously analyzed with the NON338 probe as a negative control for non-specific binding. According to Amann et al [40], probe sequences and hybridization conditions are presented in Table S1. Oligonucleotide probes were synthesized by Metabion (Martinsried, Munich, Germany). The number of bacterial cells in each wastewater and river water sample was calculated based on 20 randomly selected fields across the entire surface of the examined filter fragments, and it was expressed per mL of wastewater and river water samples.

### 2.4. Quantitative Analyses of Gene Prevalence

The conserved regions of the 16S rRNA gene and genes specific to *E. coli* (gene encoding the beta-glucosidase enzyme, *uid*A), *E. faecalis* (fragment of the 16S rRNA gene, *Faecalis*1), and BFG (gene encoding bacterioferritin, *bfr*, and a fragment of the 16S rRNA gene-HF183/BacR287) were identified in samples of genomic DNA by real-time quantitative polymerase chain reaction (qPCR). The concentrations of genes encoding resistance to five groups of antibiotics, including beta-lactams (*cfx*A, *bla*_AMP-C_), tetracyclines (*tet*(Q), *tet*(X)), macrolides, lincosamides and streptogramins (*erm*F, *mef*A, *lin*A), chloramphenicol (*cat*A1, *fex*A), and vancomycin (*van*A), were determined. The abundance of the gene responsible for the synthesis of multidrug efflux transporter pumps (*bex*A) and genes encoding class 1 and class 2 integrases (*int*I1 and *int*I2) was also determined. The copy numbers of the examined genes were expressed per mL of wastewater or river water. The qPCR protocols were optimized based on previously described primers, and are presented in Table S1 [15,16,17,41,42,43,44,45,46,47,48,49,50]. All qPCR assays were carried out in the Roche LightCycler^®^ 480 (Roche Applied Science, Indianapolis, IN, USA) in 15 µL of the reaction mix containing 1 µL (20 ng) of the genomic DNA matrix. All analyses were performed in triplicate. The standard curves for every gene were derived from serial solutions of plasmids containing the target genes.

### 2.5. Statistical Analysis

The differences in the concentrations of the analyzed genes in wastewater and river water samples were determined by Kruskal–Wallis (KW) ANOVA. The correlations between the numbers of the examined genes were determined based on the values of Spearman’s rank correlation coefficient. The Mann–Whitney U (M–W U) test for two independent samples was used to compare gene concentrations in samples of river water collected upstream (URW) and downstream (DRW) from the wastewater discharge point. Statistical analyses were conducted in the Statistica 13.2 program (StatSoft Inc., 1984–2019, Tulsa, OK, USA) at a significance level of *p* < 0.05. A cluster analysis was performed with the use of Ward’s method. The results of the cluster analysis and correlation analysis were visualized in the R environment (R v. 3.5.2 and RStudio v. 1.1.463, Boston, MA, USA) with the use of gplots and corrplot packages.

## 3. Results and Discussion

### 3.1. Total Number of Bacterial Cells and 16S rRNA Gene Copy Numbers in Wastewater and River Water Samples

**Figure 1:**
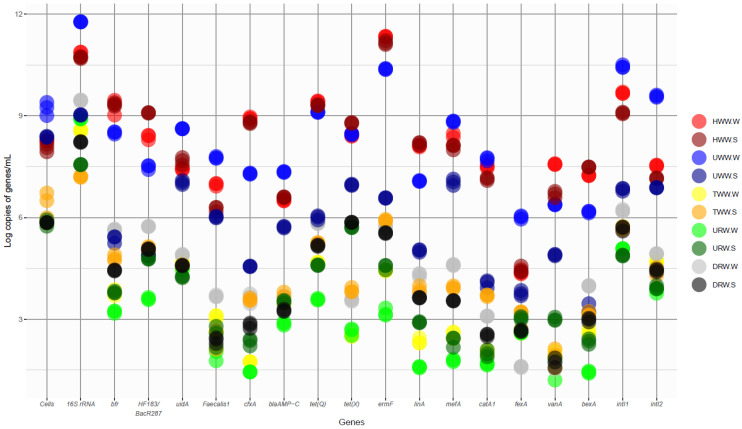
The total number of bacterial cells (FISH) and the concentrations of bacterial genes (qPCR) in wastewater and river water samples.

The TCN of the examined wastewater and river water samples ranged from 10^5^ to 10^9^ cells/mL (Figure 1). The highest values were noted in HWW (10^8^ cells/mL) and UWW (10^8^–10^9^ cells/mL). In the remaining samples, the TCN ranged from 10^5^ to 10^6^ cells/mL. The copy numbers of the 16S rRNA gene were determined by qPCR. The results were used to estimate total bacterial counts in the analyzed samples. The highest concentration of the 16S rRNA gene was noted in HWW (10^10^ copies/mL) and UWW (10^9^–10^11^ copies/mL) (Figure 1, Table S2). The abundance of the 16S rRNA gene was determined at 10^7^–10^8^ copies/mL in TWW, and at 10^8^–10^9^ copies/mL in river water sampled upstream (URW) and downstream (DRW) from the wastewater discharge point. Niestepski et al. [13], Korzeniewska and Harnisz [28], and Caucci et al. [51] reported similar 16S rRNA gene copy numbers in various samples collected from WWTPs. The TCN determined in the FISH assay was significantly lower than the concentration of the 16S rRNA gene determined by qPCR in all samples collected both in winter and summer (KW, *p* < 0.05) (Table S5). These differences can probably be attributed to the fact that a single bacterial cell can contain more than one copy of the 16S rRNA gene, and that the number of copies can differ across and within taxa [52,53]. In the FISH method, an EUB probe is used to observe and count individual bacteria regardless of the number of 16S rRNA gene copies inside each bacterial cell. The qPCR assay supports estimations of the number of 16S rRNA gene copies in a sample, but not in bacterial populations in the examined samples, and its results cannot be used to determine the exact number of bacterial cells in a sample [52]. Therefore, the FISH method appears to be better suited for determinations of TCN than qPCR.

### 3.2. Concentrations of Genes Specific to Escherichia coli, Enterococcus faecalis, and BFG, and ARGs in Wastewater and River Water Samples

**Figure 2:**
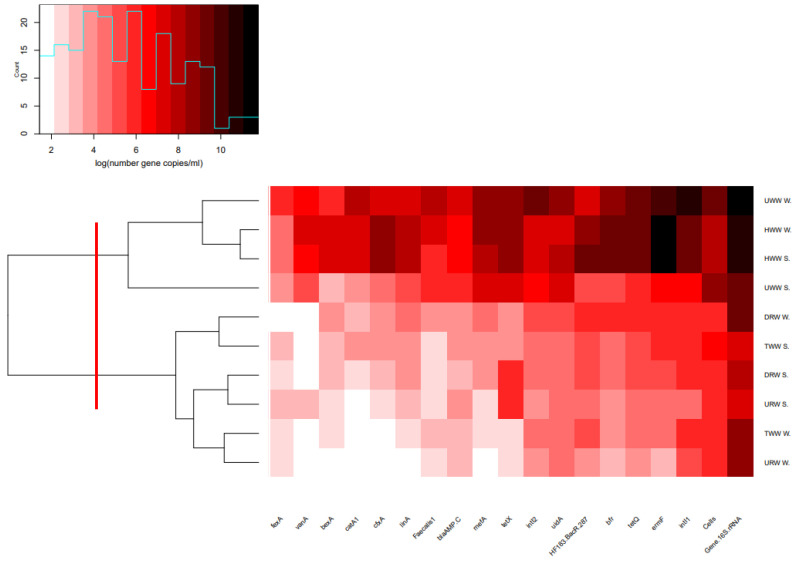
Heatmap of gene concentrations in wastewater and river water samples collected in winter (W) and summer (S) (copies/mL) (clusters are separated by the red line).

The concentrations of genes specific to indicator bacteria and BFG as well as ARGs and genes encoding integrase in samples of wastewater and river water are presented in Figure 1 and Table S2. The copy numbers of genes specific to indicator bacteria, ARGs, and integrase genes were expressed in absolute values (copies/mL), due to variations in the structure of bacterial populations in wastewater and river water samples [52], as well as differences between the TCN determined in the FISH assay and the number of 16S rRNA gene copies determined by qPCR. The absolute and relative abundance of the examined genes in each sample is presented in Figure 1 and Figure 2, and in Supplementary Materials (Tables S2–S4).

The *uid*A and *Faecalis*1 genes, which are specific to *E. coli* and *E. faecalis* fecal indicator bacteria, respectively, were identified, and the concentrations of the genes characteristic of BFG (*bfr* for *B. thetaiotaomicron*, *B. vulgatus*, *B. fragilis*, *B. caccae*, *B. ovatus*, *B. eggerthii*, *B. uniformis*, *B. stercoris*, *Parabacteroides merdae*, and *P. distasonis*; HF183/BacR287 marker for *B. dorei*) were determined in the present study. In HWW and UWW samples, the number of *uid*A and *Faecalis*1 gene copies was determined at 10^7^–10^8^ copies/mL and 10^6^–10^7^ copies/mL, respectively, whereas the abundance of genes specific to BFG ranged from 10^4^ to 10^9^ copies/mL in the examined samples. The copy numbers of the *bfr* gene and the HF183/BacR287 marker were significantly lower in UWW than in HWW (M–W U, *p* < 0.05), and their concentrations exceeded those of *uid*A and *Faecalis*1 genes in HWW. In samples of TWW and river water, the concentrations of genes specific to these bacteria were determined at 10^2^ to 10^5^ copies/mL, and the number of *Faecalis*1 gene copies was lowest (Figure 1, Table S2). The concentrations of genes specific to BFG, *E. coli*, and *E. faecalis* differed across sampling seasons (KW, *p* < 0.05) (Table S5). In all samples, the abundance of genes characteristic of *E. coli* and *E. faecalis* was below the TCN values determined in the FISH assay. In turn, the copy numbers of genes specific to BFG exceeded the TCN in HWW samples collected in both winter and summer (Table S3). In the remaining samples, the concentrations of genes specific to BFG were lower than the TCN determined by the FISH method. The *bfr* gene and the HF183/BacR287 marker are localized within 16S rRNA. The genome of a single bacterium of the genus *Bacteroides* harbors five copies of the 16S rRNA gene on average [53]. However, the results of this study point to a dominance of BFG in HWW, and to high concentrations of genes of all indicator bacteria in the remaining samples of wastewater and river water, in particular DRW. Rocha et al. [54] reported that genes characteristic of *E. coli* and *E. faecalis* are not effectively removed during wastewater treatment. Our previous studies [13,24] demonstrated that BFG bacteria are not completely eliminated in WWTPs during the activated sludge process. Feng et al. [55] and Ordaz et al. [56] argued that, similarly to *E. coli* and *E. faecalis*, *Bacteroides* species should be regarded as fecal indicator bacteria to accurately describe environmental contamination with human feces. The present findings suggest that markers specific to BFG, in particular HF183/BacR287, are not only as effective as the standard indicators of fecal contamination, but also support accurate identification of the sources of human-caused pollution, which validates the first research hypothesis.

Eleven ARGs and two genes encoding integrase synthesis were identified in the analyzed samples of wastewater and river water. Significant differences were noted between the total concentrations of ARGs and *int*I genes in winter and summer samples (KW, *p* < 0.05) (Table S5). The highest copy numbers of ARGs were determined in HWW, followed by UWW. In those samples, the concentrations of ARGs ranged from 10^3^ to 10^11^ copies/mL. Genes encoding resistance to tetracyclines (*tet*(Q), *tet*(X)), MLS antibiotics (*erm*F, *lin*A, *mef*A), and the *cfx*A gene encoding resistance to beta-lactams were most abundant, and their average concentrations in both seasons ranged from 10^7^ to 10^9^ copies/mL. The *fex*A gene encoding resistance to chloramphenicol was the least abundant ARG in HWW and UWW, and its concentration was determined at 10^3^ to 10^6^ copies/mL. The analyzed ARGs were less abundant in TWW and river water, at 10^1^ to 10^5^ copies/mL; *erm*F, *tet*(Q), and *tet*(X) were the dominant genes in those samples. The copy numbers of nearly all analyzed ARGs were lowest in URW samples. Similarly to this study, numerous researchers have reported on the widespread presence of ARGs in both TWW and the receiving water bodies [29,51,54,57,58,59,60,61,62,63]. Mao et al. [61] observed an increase in the copy numbers of ARGs during wastewater treatment in WWTPs. High concentrations of ARGs in wastewater and river water could point to the presence of drug-resistant and multidrug-resistant bacterial strains in wastewater and river water.

The concentrations of most ARGs in HWW were similar in both sampling seasons. In UWW, the copy numbers of ARGs were markedly lower in summer than in winter. According to Guo et al. [64] and Rodriguez-Mozaz et al. [65], the copy numbers of ARGs are closely correlated with antibiotic concentrations in wastewater. The results of the current study suggest that antibiotic consumption in hospitals was fairly similar in summer and winter, but it was higher in the outpatient setting in winter. Similar observations were made by Ciszewski et al. [66], who reported a significant increase in antibiotic consumption in Poland in fall/winter.

In HWW and UWW, the concentration of the *int*I1 gene ranged from 10^6^ to 10^10^ copies/mL, and the number of *int*I2 gene copies was estimated at 10^6^ to 10^9^ copies/mL. The abundance of *int*I1 and *int*I2 genes was determined at 10^3^–10^7^ copies/mL in the remaining samples. The abundance of the *int*I1 gene was considerably higher than the concentration of the *int*I2 gene. The present results corroborate the findings of other authors who reported on the dominance of the *int*I1 gene among integrase-encoding genes in environmental samples [67]. Integrons are ubiquitous in TWW [25,32,68,69] and in rivers such as the Vistula, Warta, and Łyna [29,60,70,71]. These genetic structures promote rapid bacterial evolution by enabling bacteria to accumulate, express, and transfer coding sequences such as ARGs [67,72]. According to Gillings et al. [37], class 1 integrons could be the main mobile genetic elements responsible for the spread of antibiotic resistance, due to their widespread prevalence in the environment.

The concentrations of the analyzed genes in wastewater and river water samples collected in winter and summer were subjected to cluster analysis with the use of Ward’s method. The results were visualized in a heatmap with dendrograms (Figure 2). Based on these findings, wastewater and river water samples were divided into two clusters. The first cluster was composed solely of untreated wastewater (HWW and UWW) collected in both winter and summer. These samples were characterized by the highest abundance of the tested genes. The second cluster contained samples of treated wastewater and river water (TWW, URW and DRW).

**Figure 3:**
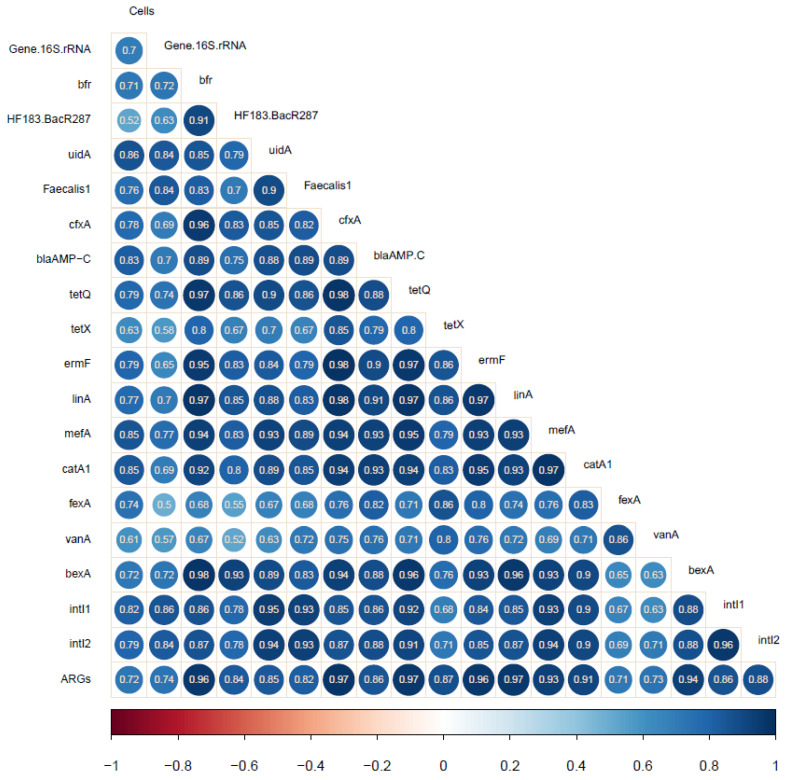
Spearman’s rank correlations between the concentrations of the analyzed genes (*p* < 0.05). Positive correlations are marked in blue, and negative correlations are marked in red. Color intensity and the size of circles correspond to the values of correlation coefficients.

A correlation matrix based on the values of Spearman’s rank correlation coefficient revealed significant relationships between the TCN, the abundance of the 16S rRNA gene, genes specific to BFG, *E. coli*, and *E. faecalis*, integrase genes, and ARGs in wastewater and river water samples (Figure 3, Table S6). The TCN was correlated with the abundance of the conserved regions of the 16S rRNA gene at r = 0.70 (*p* < 0.05). This observation confirms that despite differences in TCN and the concentration of the 16S rRNA gene in the examined environmental samples, the above values were highly correlated. Strong positive correlations were noted between the TCN and the abundance of all analyzed ARGs (r = 0.61–0.85, *p* < 0.05), which points to the presence of ARB in wastewater and river water samples. The copy numbers of genes encoding class 1 and class 2 integrons (r = 0.84–0.86, *p* < 0.05) and ARGs (r = 0.50–0.77, *p* < 0.05) increased with a rise in the concentration of the 16S rRNA gene, which could point to horizontal gene transfer between bacterial populations colonizing wastewater and river water. Similar results were reported by An et al. [67].

The prevalence of genes specific to BFG in the examined samples was closely correlated with the concentrations of genes characteristic of *E. coli* (*uid*A) and *E. faecalis* (*Faecalis*1) (r = 0.70–0.85, *p* < 0.05). These results indicate that BFG bacteria coexist with *E. coli* and *E. faecalis*, and should be regarded as microbial indicators of water quality in screening tests. The abundance of genes specific to BFG, *E. coli*, and *E. faecalis* was also highly correlated with all examined ARGs (r = 0.52–0.98, *p* < 0.05). The presence of indicator bacteria in wastewater and river water samples points to the coexistence of ARGs in the analyzed environments, and high concentrations of ARGs in water can be attributed to contamination with indicator bacteria [73,74]. In the present study, a significant correlation was also noted between the copy numbers of *van*A and *Faecalis*1 genes (r = 0.72, *p* < 0.05), which could suggest that vancomycin-resistant *E. faecalis* strains (VRE) were present in the tested samples. In a study by Oravcova et al. [75], enterococci harboring the *van*A gene were frequently identified in TWW, which indicates that these bacteria are not effectively eliminated during wastewater treatment. The high coefficients of correlation between the abundance of genes specific to BFG and genes encoding resistance to tetracyclines, MLS antibiotics, beta-lactams, and the *bex*A gene confirm previous findings that BFG bacteria are a major reservoir of these ARGs [24,76,77]. In the current study, close correlations were found between the prevalence of all analyzed ARGs in environmental samples (r = 0.63–0.98, *p* < 0.05). These results, as well as previous findings [29], point to the presence of correlations between the abundance of all examined ARGs in HWW, UWW, TWW, and river water. The genes specific to BFG are not only indicators of fecal pollution, but they can also be used to determine the spread of ARGs in the environment. Karkman et al. [73] and Stachler et al. [78] also demonstrated that the presence of ARGs in the environment is closely related to fecal contamination markers, and therefore it could be associated with fecal contamination of the environment.

### 3.3. The Influence of WWTPs on Gene Abundance in River Water

The abundance of the analyzed genes in samples of river water collected upstream and downstream from the wastewater discharge point (URW and DRW, respectively) was compared to determine the potential influence of a WWTP on the contamination of river water, and to evaluate the applicability of markers specific to BFG bacteria as indicators of anthropogenic pollution (Table S7). In the group of genes specific to BFG, *E. coli*, and *E. faecalis*, a significant increase in the copy numbers of *bfr* and *uid*A genes and the HF183/BacR287 marker was noted in DRW (M-W U, *p* < 0.05), whereas no differences were observed in the number of *Faecalis*1 gene copies. These results clearly indicate that the evaluated WWTP contributed to the fecal contamination of river water. The concentrations of seven out of the 11 (63.63%) tested ARGs were higher in DRW than in URW (M–W U, *p* < 0.05). The abundance of selected genes was three orders of magnitude higher in DRW than in URW, and the greatest differences were observed in winter. No significant differences in the abundance of the remaining four genes (*bla*_AMP-C_, *tet*(Q), *fex*A, and *van*A) were noted in river water samples (M–W U, *p* > 0.05). The copy numbers of both genes encoding integrase synthesis were higher in DRW than in URW (M–W U, *p* > 0.05). These results indicate, in accordance with the second research hypothesis, that WWTPs contribute to the contamination of river water with genes specific to indicator bacteria and BFG as well as ARGs and genes encoding class 1 and class 2 integrases. Numerous researchers have demonstrated that WWTPs are important sources of multidrug-resistant bacteria, including bacteria that are potentially pathogenic for humans (*B. fragilis*, *E. coli*, and *E. faecalis*) and harbor integrons or gene cassettes that carry resistance genes [24,28,29,67,79,80]. According to Giebułtowicz et al. [79], these observations can be probably attributed to the fact that wastewater is only partially treated in WWTPs. Environmental contamination with indicator bacteria can be detected with qPCR-based techniques, which offer a viable alternative to standard time-consuming culture methods [81,82]. The qPCR methods also support the use of specific indicators, such as the *bfr* gene and the HF183/BacR287 marker, in environmental screening tests, to determine fecal contamination and human-caused pollution, including the presence of ARGs.

## 4. Conclusions

This study evaluated the applicability of markers specific to BFG bacteria as indicators of anthropogenic pollution in surface waters. Samples of HWW, UWW, TWW, and water from a river receiving TWW were analyzed. The concentrations of genes specific to BFG were high in all samples, and they were closely correlated with the abundance of genes specific to *E. coli* and *E. faecalis*, as well as ARGs and integrase genes. These results suggest that genetic markers specific to BFG can be used as indicators of anthropogenic pollution in the aquatic environment. The present findings indicate that the presence of indicator bacteria in wastewater and river water samples is correlated with the abundance of ARGs in these environments. This study also demonstrated that TWW evacuated from the examined WWTP contributes to the contamination of river water with genes specific to fecal bacteria, ARGs, and genes encoding class 1 and class 2 integrases.

## Supplementary Materials

The following are available online at https://www.mdpi.com/1660-4601/17/19/7137/s1: Figure S1. Genes copy number per cells in environmental samples; Figure S2. Cells and genes copy number per 16S rRNA in environmental samples; Table S1. Oligonucleotide primers and parameters used for the detection of genes, with qPCR analysis and FISH; Table S2. Average gene concentrations in wastewater and river water samples (copies/mL); Table S3. Average gene concentrations in wastewater and river water samples (copies/cells); Table S4. Average gene concentrations in wastewater and river water samples (kopii/16S rRNA); Table S5. Differences in gene concentrations in the analyzed samples between seasons (Kruskal–Wallis ANOVA; significant results are marked in red, *p* < 0.05); Table S6. Correlations between gene concentrations (Spearman’s rank correlation coefficient; significant results are marked in red, *p* < 0.05); Table S7. A comparison of gene concentrations in URW and DRW (Mann–Whitney U test; significant results are marked in red, *p* < 0.05).

## References

[1] (2014). ISO 9308-1:2014/AMD 1:2016, Water Quality—Enumeration of Escherichia Coli and Coliform Bacteria—Part 1: Membrane Filtration Method for Waters with Low Bacterial Background Flora—Amendment 1.

[2] (2012). ISO 9308-2:2012, Water Quality—Enumeration of Escherichia Coli and Coliform Bacteria—Part 2: Most Probable Number Method.

[3] (2000). ISO 7899-1:1998/COR 1:2000, Water Quality—Detection and Enumeration of Intestinal Enterococci—Part 1: Miniaturized Method (Most Probable Number) for Surface and Waste Water—Technical Corrigendum 1.

[4] (2000). ISO 7899-2:2000, Water Quality—Detection and Enumeration of Intestinal Enterococci—Part 2: Membrane Filtration Method.

[5] Boehm A.B., Ashbolt N.J., Colford J.M., Dunbar L.E., Fleming L.E., Gold M.A., Hansel A., Hunter P.R., Ichida A.M., McGee C.D. (2009). A sea change ahead for recreational water quality criteria. J. Water Health.

[6] Schriewer A., Odagiri M., Wuertz S., Misra P.R., Panigrahi P., Clasen T., Jenkins M.W. (2015). Human and Animal Fecal Contamination of Community Water Sources, Stored Drinking Water and Hands in Rural India Measured with Validated Microbial Source Tracking Assays. Am. J. Trop. Med. Hyg..

[7] Byappanahalli M.N., Nevers M.B., Korajkic A., Staley Z.R., Harwood V.J. (2012). Enterococci in the Environment. Microbiol. Mol. Biol. Rev..

[8] Gomez-Donate M., Casanovas-Massana A., Muniesa M., Blanch A.R. (2016). Development of new host-specific Bacteroides qPCRs for the identification of fecal contamination sources in water. MicrobiologyOpen.

[9] Cao Y.P., Sivaganesan M., Kelty C.A., Wang D., Boehm A.B., Griffith J.F., Weisberg S.B., Shanks O.C. (2018). A human fecal contamination score for ranking recreational sites using the HF183/BacR287 quantitative real-time PCR method. Water Res..

[10] Harwood V.J., Staley C., Badgley B.D., Borges K., Korajkic A. (2014). Microbial source tracking markers for detection of fecal contamination in environmental waters: Relationships between pathogens and human health outcomes. FEMS Microbiol. Rev..

[11] Parker J.K., McIntyre D., Noble R.T. (2010). Characterizing fecal contamination in stormwater runoff in coastal North Carolina, USA. Water Res..

[12] Sercu B., Van De Werfhorst L.C., Murray J., Holden P.A. (2009). Storm Drains are Sources of Human Fecal Pollution during Dry Weather in Three Urban Southern California Watersheds. Environ. Sci. Technol..

[13] Niestępski S., Harnisz M., Ciesielski S., Korzeniewska E., Osińska A. (2020). Environmental fate of Bacteroidetes, with particular emphasis on Bacteroides fragilis group bacteria and their specific antibiotic resistance genes, in activated sludge wastewater treatment plants. J. Hazard. Mater..

[14] Wu G.D., Chen J., Hoffmann C., Bittinger K., Chen Y.Y., Keilbaugh S.A., Bewtra M., Knights D., Walters W.A., Knight R. (2011). Linking Long-Term Dietary Patterns with Gut Microbial Enterotypes. Science.

[15] Bernhard A.E., Field K.G. (2000). A PCR assay to discriminate human and ruminant feces on the basis of host differences in Bacteroides-Prevotella genes encoding 16S rRNA. Appl. Environ. Microbiol..

[16] Green H.C., Haugland R.A., Varma M., Millen H.T., Borchardt M.A., Field K.G., Walters W.A., Knight R., Sivaganesan M., Kelty C.A. (2014). Improved HF183 Quantitative Real-Time PCR Assay for Characterization of Human Fecal Pollution in Ambient Surface Water Samples. Appl. Environ. Microbiol..

[17] Liu C., Song Y., McTeague M., Vu A.W., Wexler H., Finegold S.M. (2003). Rapid identification of the species of the Bacteroides fragilis group by multiplex PCR assays using group-and species-specific primers. FEMS Microbiol. Lett..

[18] WHO (2014). Antimicrobial Resistance: Global Report on Surveillance 2014.

[19] Zhang Q.Q., Ying G.G., Pan C.G., Liu Y.S., Zhao J.L. (2015). Comprehensive Evaluation of Antibiotics Emission and Fate in the River Basins of China: Source Analysis, Multimedia Modeling, and Linkage to Bacterial Resistance. Environ. Sci. Technol..

[20] Wu D.L., Zhang M., He L.X., Zou H.Y., Liu Y.S., Li B.B., Yang Y.Y., Liu C.X., He L.Y., Ying G.G. (2020). Contamination profile of antibiotic resistance genes in ground water in comparison with surface water. Sci. Total Environ..

[21] Karkman A., Do T.T., Walsh F., Virta M.P.J. (2018). Antibiotic-Resistance Genes in Waste Water. Trends Microbiol..

[22] Rizzo L., Manaia C., Merlin C., Schwartz T., Dagot C., Ploy M.C., Michael I., Fatta-Kassinos D. (2013). Urban wastewater treatment plants as hotspots for antibiotic resistant bacteria and genes spread into the environment: A review. Sci. Total Environ..

[23] Allen H.K., Donato J., Wang H.H., Cloud-Hansen K.A., Davies J., Handelsman J. (2010). Call of the wild: Antibiotic resistance genes in natural environments. Nat. Rev. Microbiol.

[24] Niestepski S., Harnisz M., Korzeniewska E., Aguilera-Arreola M.G., Contreras-Rodriguez A., Filipkowska Z., Osinska A. (2019). The emergence of antimicrobial resistance in environmental strains of the Bacteroides fragilis group. Environ. Int..

[25] Marin I., Goni P., Lasheras A.M., Ormad M.P. (2015). Efficiency of a Spanish wastewater treatment plant for removal potentially pathogens: Characterization of bacteria and protozoa along water and sludge treatment lines. Ecol. Eng..

[26] Bengtsson-Palme J., Kristiansson E., Larsson D.G.J. (2018). Environmental factors influencing the development and spread of antibiotic resistance. FEMS Microbiol. Rev..

[27] Huang J.J., Hu H.Y., Lu S.Q., Li Y., Tang F., Lu Y., Wei B. (2012). Monitoring and evaluation of antibiotic-resistant bacteria at a municipal wastewater treatment plant in China. Environ. Int..

[28] Korzeniewska E., Harnisz M. (2018). Relationship between modification of activated sludge wastewater treatment and changes in antibiotic resistance of bacteria. Sci. Total Environ..

[29] Osinska A., Korzeniewska E., Harnisz M., Felis E., Bajkacz S., Jachimowicz P., Niestepski S., Konopka I. (2020). Small-scale wastewater treatment plants as a source of the dissemination of antibiotic resistance genes in the aquatic environment. J. Hazard. Mater..

[30] Yang Y., Li B., Zou S.C., Fang H.H.P., Zhang T. (2014). Fate of antibiotic resistance genes in sewage treatment plant revealed by metagenomic approach. Water Res..

[31] Czekalski N., Berthold T., Caucci S., Egli A., Burgmann H. (2012). Increased levels of multiresistant bacteria and resistance genes after wastewater treatment and their dissemination into Lake Geneva, Switzerland. Front. Microbiol..

[32] Zhang Y.L., Marrs C.F., Simon C., Xi C.W. (2009). Wastewater treatment contributes to selective increase of antibiotic resistance among Acinetobacter spp.. Sci. Total Environ..

[33] Osinska A., Harnisz M., Korzeniewska E. (2016). Prevalence of plasmid-mediated multidrug resistance determinants in fluoroquinolone-resistant bacteria isolated from sewage and surface water. Environ. Sci. Pollut. Res..

[34] Osinska A., Korzeniewska E., Harnisz M., Niestepski S. (2017). The prevalence and characterization of antibiotic-resistant and virulent Escherichia coli strains in the municipal wastewater system and their environmental fate. Sci. Total Environ..

[35] Qiao M., Ying G.G., Singer A.C., Zhu Y.G. (2018). Review of antibiotic resistance in China and its environment. Environ. Int..

[36] Aubertheau E., Stalder T., Mondamert L., Ploy M.C., Dagot C., Labanowski J. (2017). Impact of wastewater treatment plant discharge on the contamination of river biofilms by pharmaceuticals and antibiotic resistance. Sci. Total Environ..

[37] Gillings M.R. (2014). Integrons: Past, Present, and Future. Microbiol. Mol. Biol. Rev..

[38] Gillings M.R., Gaze W.H., Pruden A., Smalla K., Tiedje J.M., Zhu Y.G. (2015). Using the class 1 integron-integrase gene as a proxy for anthropogenic pollution. ISME J..

[39] Zheng W.L., Huyan J.Q., Tian Z., Zhang Y., Wen X.H. (2020). Clinical class 1 integron-integrase gene—A promising indicator to monitor the abundance and elimination of antibiotic resistance genes in an urban wastewater treatment plant. Environ. Int..

[40] Amann R.I., Binder B.J., Olson R.J., Chisholm S.W., Devereux R., Stahl D.A. (1990). Combination of 16S rRNA-targeted oligonucleotide probes with flow cytometry for analyzing mixed microbial populations. Appl. Environ. Microbiol..

[41] Nadkarni M.A., Martin F.E., Jacques N.A., Hunter N. (2002). Determination of bacterial load by real-time PCR using a broad-range (universal) probe and primers set. Microbiology.

[42] Heijnen L., Medema G. (2006). Quantitative detection of *E. coli*, *E. coli* O157 and other shiga toxin producing *E. coli* in water samples using a culture method combined with real-time PCR. J. Water Health.

[43] Lu J., Santo Domingo J.W., Lamendella R., Edge T., Hill S. (2008). Phylogenetic diversity and molecular detection of bacteria in gull feces. Appl. Environ. Microbiol..

[44] Eitel Z., Soki J., Urban E., Nagy E., Anaerobic E.S.G. (2013). The prevalence of antibiotic resistance genes in Bacteroides fragilis group strains isolated in different European countries. Anaerobe.

[45] Ruppé E., Hem S., Lath S., Gautier V., Ariey F., Sarthou J., Monchy D., Arlet G. (2009). CTX-M β-lactamases in Escherichia coli from community-acquired urinary tract infections.” Cambodia. Emerg. Infect. Dis..

[46] Ng L.K., Martin I., Alfa M., Mulvey M. (2001). Multiplex PCR for the detection of tetracycline resistant genes. Mol. Cell. Probes.

[47] Li J., Shao B., Shen J., Wang S., Wu Y. (2013). Occurrence of Chloramphenicol-Resistance Genes as Environmental Pollutants from Swine Feedlots. Environ. Sci. Technol..

[48] Maidhof H., Guerra B., Abbas S., Elsheikha H.M., Whittam T.S., Beutin L. (2002). A multiresistant clone of Shiga toxin-producing Escherichia coli O118: H16 is spread in cattle and humans over different European countries. Appl. Environ. Microbiol..

[49] He Y.H., Ruan G.J., Hao H., Xue F., Ma Y.K., Zhu S.N., Zheng B. (2019). Real-time PCR for the rapid detection of vanA, vanB and vanM genes. J. Microbiol. Immunol. Infect..

[50] Goldstein C., Lee M.D., Sanchez S., Hudson C., Phillips B., Register B., Grady M., Liebert C., Summers A.O., White D.G. (2001). Incidence of class 1 and 2 integrases in clinical and commensal bacteria from livestock, companion animals, and exotics. Antimicrob. Agents Chemother..

[51] Caucci S., Karkman A., Cacace D., Rybicki M., Timpel P., Voolaid V., Gurke R., Virta M., Berendonk T.U. (2016). Seasonality of antibiotic prescriptions for outpatients and resistance genes in sewers and wastewater treatment plant outflow. FEMS Microbiol. Ecol..

[52] Kembel S.W., Wu M., Eisen J.A., Green J.L. (2012). Incorporating 16S Gene Copy Number Information Improves Estimates of Microbial Diversity and Abundance. PLoS Comput. Biol..

[53] Vetrovsky T., Baldrian P. (2013). The Variability of the 16S rRNA Gene in Bacterial Genomes and Its Consequences for Bacterial Community Analyses. PLoS ONE.

[54] Rocha J., Fernandes T., Riquelme M.V., Zhu N., Pruden A., Manaia C.M. (2019). Comparison of Culture- and Quantitative PCR-Based Indicators of Antibiotic Resistance in Wastewater, Recycled Water, and Tap Water. Int. J. Environ. Res. Public Health.

[55] Feng S.C., McLellan S.L. (2019). Highly Specific Sewage-Derived Bacteroides Quantitative PCR Assays Target Sewage-Polluted Waters. Appl. Environ. Microbiol..

[56] Ordaz G., Merino-Mascorro J.A., Garcia S., Heredia N. (2019). Persistence of Bacteroidales and other fecal indicator bacteria on inanimated materials, melon and tomato at various storage conditions. Int. J. Food Microbiol..

[57] Chen B.A., Hao L.J., Guo X.Y., Wang N., Ye B.P. (2015). Prevalence of antibiotic resistance genes of wastewater and surface water in livestock farms of Jiangsu Province, China. Environ. Sci. Pollut. Res..

[58] Fan X.Y., Gao J.F., Pan K.L., Li D.C., Dai H.H., Li X. (2018). Functional genera, potential pathogens and predicted antibiotic resistance genes in 16 full-scale wastewater treatment plants treating different types of wastewater. Bioresour. Technol..

[59] Jia S.Y., Zhang X.X., Miao Y., Zhao Y.T., Ye L., Li B., Zhang T. (2017). Fate of antibiotic resistance genes and their associations with bacterial community in livestock breeding wastewater and its receiving river water. Water Res..

[60] Makowska N., Koczura R., Mokracka J. (2016). Class 1 integrase, sulfonamide and tetracycline resistance genes in wastewater treatment plant and surface water. Chemosphere.

[61] Mao D.Q., Yu S., Rysz M., Luo Y., Yang F.X., Li F.X., Hou J., Mu Q.H., Alvarez P.J.J. (2015). Prevalence and proliferation of antibiotic resistance genes in two municipal wastewater treatment plants. Water Res..

[62] Narciso-da-Rocha C., Manaia C.M. (2017). The influence of the autochthonous wastewater microbiota and gene host on the fate of invasive antibiotic resistance genes. Sci. Total Environ..

[63] Wang M.Y., Shen W.T., Yan L., Wang X.H., Xu H. (2017). Stepwise impact of urban wastewater treatment on the bacterial community structure, antibiotic contents, and prevalence of antimicrobial resistance. Environ. Pollut..

[64] Guo X.Y., Yan Z., Zhang Y., Xu W.L., Kong D.Y., Shan Z.J., Wang N. (2018). Behavior of antibiotic resistance genes under extremely high-level antibiotic selection pressures in pharmaceutical wastewater treatment plants. Sci. Total Environ..

[65] Rodriguez-Mozaz S., Chamorro S., Marti E., Huerta B., Gros M., Sanchez-Melsio A., Borrego C.M., Barcelo D., Balcazar J.L. (2015). Occurrence of antibiotics and antibiotic resistance genes in hospital and urban wastewaters and their impact on the receiving river. Water Res..

[66] Ciszewski M., Czekaj T., Szewczyk E.M. (2017). Outpatient Antibiotic Consumption Fluctuations in a View of Unreasonable Antibacterial Therapy. Pol. J. Microbiol..

[67] An X.L., Chen Q.L., Zhu D., Zhu Y.G., Gillings M.R., Su J.Q. (2018). Impact of Wastewater Treatment on the Prevalence of Integrons and the Genetic Diversity of Integron Gene Cassettes. Appl. Environ. Microbiol..

[68] Zhang X.X., Zhang T., Zhang M., Fang H.H.P., Cheng S.P. (2009). Characterization and quantification of class 1 integrons and associated gene cassettes in sewage treatment plants. Appl. Microbiol. Biotechnol..

[69] Lin M., Liang J.J., Zhang X., Wu X.M., Yan Q.P., Luo Z.X. (2015). Genetic diversity of three classes of integrons in antibiotic-resistant bacteria isolated from Jiulong River in southern China. Environ. Sci. Pollut. Res..

[70] Koczura R., Mokracka J., Jablonska L., Gozdecka E., Kubek M., Kaznowski A. (2012). Antimicrobial resistance of integron-harboring Escherichia coli isolates from clinical samples, wastewater treatment plant and river water. Sci. Total Environ..

[71] Kotlarska E., Luczkiewicz A., Pisowacka M., Burzynski A. (2015). Antibiotic resistance and prevalence of class 1 and 2 integrons in Escherichia coli isolated from two wastewater treatment plants, and their receiving waters (Gulf of Gdansk, Baltic Sea, Poland). Environ. Sci. Pollut. Res..

[72] Escudero J.A., Loot C., Mazel D. (2018). Integrons as adaptive devices. Molecular Mechanisms of Microbial Evolution.

[73] Karkman A., Parnanen K., Larsson D.G.J. (2019). Fecal pollution can explain antibiotic resistance gene abundances in anthropogenically impacted environments. Nat. Commun..

[74] Li L.G., Yin X.L., Zhang T. (2018). Tracking antibiotic resistance gene pollution from different sources using machine-learning classification. Microbiome.

[75] Oravcova V., Mihalcin M., Zakova J., Pospisilova L., Masarikova M., Literak I. (2017). Vancomycin-resistant enterococci with vanA gene in treated municipal wastewater and their association with human hospital strains. Sci. Total Environ..

[76] Roberts M.C. (2003). Acquired tetracycline and/or macrolide-lincosamides-streptogramin resistance in anaerobes. Anaerobe.

[77] Volkers G., Damas J.M., Palm G.J., Panjikar S., Soares C.M., Hinrichs W. (2013). Putative dioxygen-binding sites and recognition of tigecycline and minocycline in the tetracycline-degrading monooxygenase TetX. Acta Cryst. D Biol. Crystallogr..

[78] Stachler E., Crank K., Bibby K. (2019). Co-occurrence of crAssphage with antibiotic resistance genes in an impacted urban watershed. Environ. Sci. Technol. Lett..

[79] Giebultowicz J., Tyski S., Wolinowska R., Grzybowska W., Zareba T., Drobniewska A., Wroczynski P., Nalecz-Jawecki G. (2018). Occurrence of antimicrobial agents, drug-resistant bacteria, and genes in the sewage-impacted Vistula River (Poland). Environ. Sci. Pollut. Res..

[80] Tennstedt T., Szczepanowski R., Braun S., Puhler A., Schluter A. (2003). Occurrence of integron-associated resistance gene cassettes located on antibiotic resistance plasmids isolated from a wastewater treatment plant. FEMS Microbiol. Ecol..

[81] Kinzelman J., Anan’eva T., Mudd D. (2013). Evaluation of Rapid Bacteriological Analytical Methods for Use as Fecal Indicators of Beach Contamination. Report prepared for. U.S. Environmental Protection Agency, Office of Science and Technology. Region 5 Water Division, EPA Contract #EP-11-5-000072.

[82] Sivaganesan M., Aw T.G., Briggs S., Dreelin E., Asian A., Dorevitch S., Shrestha A., Isaacs N., Kinzelman J., Kleinheinz G. (2019). Standardized data quality acceptance criteria for a rapid Escherichia coli qPCR method (Draft Method C) for water quality monitoring at recreational beaches. Water Res..

