# Supplementary material for: Markers Specific to Bacteroides fragilis Group Bacteria as Indicators of Anthropogenic Pollution of Surface Waters
Source: Int J Environ Res Public Health. 2020 Sep 29;17(19):7137. doi: 10.3390/ijerph17197137 (PMC7579016; doi:10.3390/ijerph17197137)
Supplement: Supplementary file 1 [file ijerph-17-07137-s001.pdf]

Supplementary Materials

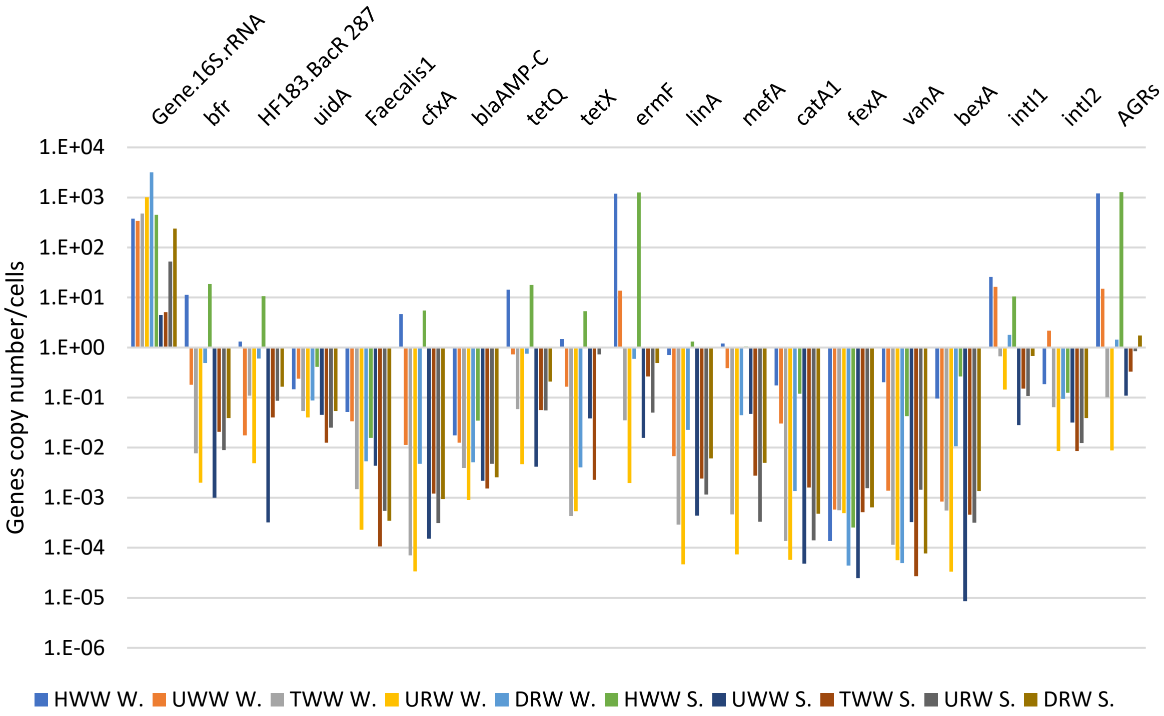

Figure S1. Genes copy number per cells in environmental samples.

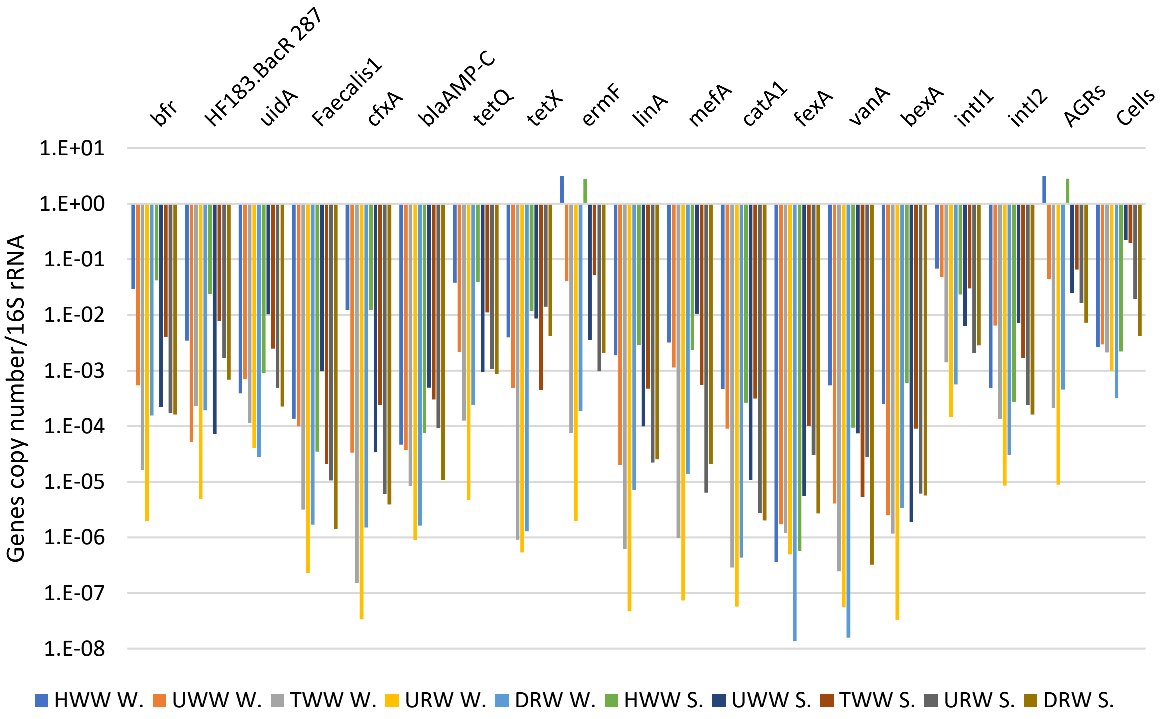

Figure S2. Cells and genes copy number per 16S rRNA in environmental samples.

Table S1. Oligonucleotide primers and parameters used for the detection of genes, with qPCR analysis and FISH.

Target Gene/Probe	Primers 5′ – 3′	Annealing Temperature in Qpcr (°C) / % FA [A] in Situ	Reference
HF183/ BacR287	ATCATGAGTTCACATGTCCG<br>CTTCCTCTCAGAACCCCTATCC	60	[15,16]
bfr	CTGAACCAGCCAAGTAGCG<br>CCGCAAACTTTCACAACTGACTTA	52	[17]
EUB338	GCT GCC TCC<br>CGT AGG AGT	35[a]	[40]
16S rRNA	TCCTACGGGAGGCAGCAGT<br>GGACTACCAGGGTATCTAATCCTGTT	56	[41]
*uid*A	ATGGAATTTCGCCGATTTTGC<br>ATTGTTTGCCTCCCTGCTGC	60	[42]
*Faecalis*1	CGCTTCTTTCCTCCCGAGT<br>GCCATGCGGCATAAACTG	60	[43]
*cfx*A	TGACTGGCCCTGAATAATCT<br>ACAAAAGATAGCGCAAATCC	55	[44]
tet(X)	TTAGCCTTACCAATGGGTGT<br>CAAATCTGCTGTTTCATTCG	55	
*erm*F	TAGATATTGGGGCAGGCAAG<br>GGAAATTGCGGAACTGCAAA	58	
*mef*A	ATACCCCAGCACTCAATTCG<br>CAATCACAGCACCCAATACG	59	
*lin*A	CTGGGGAGTGGATGTCTTGT<br>AGTTGGCTTGTTTGGAAGTG	60	
*bex*A	TAGTGGTTGCTGCGATTCTG<br>TCAGCGTCTTGGTCTGTGTC	60	
bla$_{AMP\text{-}C}$	AATGGGTTTTCTACGGTCTG<br>GGGCAGCAAATGTGGAGCAA	55	[45]
tet(Q)	TTATACTTCCTCCGGCATCG<br>ATCGGTTCGAGAATGTCCAC	55	[46]
fexA	ATTCTCCCGCAAATAACG<br>TCGGCTCAGTAGCATCACG	52	[47]
catA1	AAGTTGGCAGCATTCACCCG<br>TCGTGGTATTCACTCCAGAGCG	61	[48]
*van*A	GCCGGAAAAAGGCTCTGAA<br>TTTTTTGCCGTTTCCTGTATCC	60	[49]
*intl*1	CCTCCCGCACGATGATC<br>TCCACGCATCGTCAGGC	54	[50]
*Intl*2	TTATTGCTGGGATTAGGC<br>ACGGCTACCCTCTGTTATC	50	

[a] Percentage of formamide (FA) in in situ hybridization buffer.

Table 2. Average gene concentrations in wastewater and river water samples (copies/mL).

Season	Type of Samples	16S rRNA	bfr	HF183 / BacR 287	uidA	*Faecalis* 1	cfxA	bla_{AMP-C}	tet(Q)	tet(X)	ermF	linA	mefA	catA1	fexA	vanA	bexA	intI1	intI2	ARGs	Cells
Winter	HWW	6.92E + 10	2.05E + 09	2.40E + 08	2.70E + 07	9.46E + 06	8.55E + 08	3.23E + 06	2.62E + 09	2.71E + 08	2.17E + 11	1.30E + 08	2.21E + 08	3.18E + 07	2.48E + 04	3.74E + 07	1.74E + 07	4.73E + 09	3.38E + 07	2.21E+ 11	1.83E + 08
	UWW	5.91E + 11	3.18E + 08	3.10E + 07	4.16E + 08	5.86E + 07	1.97E + 07	2.20E + 07	1.29E + 09	2.87E + 08	2.40E + 10	1.19E + 07	6.75E + 08	5.35E + 07	1.02E + 06	2.40E + 06	1.47E + 06	2.86E + 10	3.79E + 09	2.64E+ 10	1.75E+ 09
	TWW	3.71E + 08	6.07E + 03	8.58E + 04	4.23E + 04	1.17E + 03	5.56E + 01	3.07E + 03	4.64E + 04	3.37E + 02	2.78E + 04	2.26E + 02	3.62E + 02	1.07E + 02	4.41E + 02	9.02E + 01	4.31E + 02	5.21E + 05	5.07E + 04	7.93E+ 04	7.84E+ 05
	URW	8.26E + 08	1.65E + 03	4.03E + 03	3.31E + 04	1.89E + 02	2.78E + 01	7.41E + 02	3.85E + 03	4.41E + 02	1.61E + 03	3.86E + 01	6.07E + 01	4.72E + 01	4.07E + 02	4.66E + 01	2.74E + 01	1.20E + 05	7.04E + 03	7.30E+ 03	8.23E+ 05
	DRW	2.84E + 09	4.38E + 05	5.43E + 05	7.85E + 04	4.78E + 03	4.28E + 03	4.62E + 03	6.74E + 05	3.65E + 03	5.34E + 05	2.03E + 04	3.97E + 04	1.23E + 03	3.93E + 01	4.47E + 01	9.63E + 03	1.60E + 06	8.48E + 04	1.29E+ 06	8.97E+ 05
Summer	HWW	5.20E + 10	2.16E + 09	1.22E + 09	4.72E + 07	1.81E + 06	6.30E + 08	3.98E + 06	2.05E + 09	6.17E + 08	1.44E + 11	1.52E + 08	1.23E + 08	1.38E + 07	2.93E + 04	4.90E + 06	3.07E + 07	1.21E + 09	1.44E + 07	1.48E+ 11	1.15E+ 08
	UWW	1.06E + 09	2.36E + 05	7.65E + 04	1.08E + 07	1.03E + 06	3.61E + 04	5.22E + 05	9.96E + 05	9.19E + 06	3.76E + 06	1.05E + 05	1.12E + 07	1.15E + 04	5.88E + 03	7.81E + 04	2.03E + 03	6.75E + 06	7.57E + 06	2.59E+ 07	2.38E+ 08
	TWW	1.59E + 07	6.46E + 04	1.25E + 05	3.93E + 04	3.36E + 02	3.79E + 03	4.78E + 03	1.76E + 05	7.17E + 03	8.24E + 05	7.52E + 03	8.67E + 03	4.98E + 03	1.62E + 03	8.51E + 01	1.44E + 03	4.74E + 05	2.69E + 04	1.04E+ 06	3.13E+ 06
	URW	3.63E + 07	6.23E + 03	6.03E + 04	1.76E + 04	3.83E + 02	2.17E + 02	3.33E + 03	3.91E + 04	5.13E + 05	3.50E + 04	8.09E + 02	2.33E + 02	9.91E + 01	1.09E + 03	1.01E + 03	2.23E + 02	7.54E + 04	8.60E + 03	5.94E+ 05	7.00E+ 05
	DRW	1.69E + 08	2.74E + 04	1.16E + 05	3.83E + 04	2.43E + 02	6.63E + 02	1.81E + 03	1.48E + 05	7.18E + 05	3.49E + 05	4.30E + 03	3.50E + 03	3.39E + 02	4.53E + 02	5.47E + 01	9.60E + 02	4.77E + 05	2.74E + 04	1.23E+ 06	7.06E+ 05

Table S3. Average gene concentrations in wastewater and river water samples (copies/cells).

Season	Type of Samples	16S rRNA	bfr	HF183 /BacR 287	uidA	*Faecalis* 1	cfxA	bla_{AMP-C}	tet(Q)	tet(X)	ermF	linA	mefA	catA1	fexA	vanA	bexA	intI1	intI2	ARGs
Winter	HWW	3.78E + 02	1.12 E + 01	1.31E + 00	1.48E− 01	5.17E − 02	4.6E+ 00	1.77E − 02	1.43E + 01	1.48E + 00	1.19E + 03	7.10E − 01	1.21E + 00	1.74E − 01	1.36E − 04	2.04E − 01	9.53E − 02	2.59E + 01	1.85E − 01	1.21E + 03
	UWW	3.37E + 02	1.82 E − 01	1.77E − 02	2.38E − 01	3.35E − 02	1.12E − 02	1.26E − 02	7.36E − 01	1.64E − 01	1.37E + 01	6.80E − 03	3.85E − 01	3.05E − 02	5.80E − 04	1.37E − 03	8.38E − 04	1.63E + 01	2.16E + 00	1.51E + 01

	TWW	4.73E + 02	7.75 E − 03	1.09E − 01	5.39E − 02	1.49E − 03	7.09E − 05	3.92E − 03	5.92E − 02	4.30E − 04	3.54E − 02	2.88E − 04	4.61E − 04	1.36E − 04	5.62E − 04	1.15E − 04	5.50E − 04	6.65E − 01	6.46E − 02	1.01E − 01
	URW	1.00E + 03	2.00 E − 03	4.90E − 03	4.03E − 02	2.30E − 04	3.38E − 05	9.01E − 04	4.68E − 03	5.36E − 04	1.96E − 03	4.69E − 05	7.38E − 05	5.74E − 05	4.94E − 04	5.66E − 05	3.33E − 05	1.46E − 01	8.56E − 03	8.88E − 03
	DRW	3.16E + 03	4.88 E − 01	6.05E − 01	8.75E − 02	5.33E − 03	4.77E − 03	5.15E − 03	7.51E − 01	4.07E − 03	5.95E − 01	2.27E − 02	4.43E − 02	1.37E − 03	4.38E − 05	4.98E − 05	1.07E − 02	1.79E + 00	9.45E − 02	1.44E + 00
	HWW	4.51E + 02	1.88 E + 01	1.06E + 01	4.10E − 01	1.57E − 02	5.4E + 00	3.45E − 02	1.78E + 01	5.35E + 00	1.25E + 03	1.31E + 00	1.06E + 00	1.20E − 01	2.54E − 04	4.25E − 02	2.66E − 01	1.05E + 01	1.24E − 01	1.28E + 03
	UWW	4.44E + 00	9.91 E − 04	3.21E − 04	4.54E − 02	4.32E − 03	1.51E − 04	2.19E − 03	4.18E − 03	3.85E − 02	1.58E − 02	4.39E − 04	4.70E − 02	4.80E − 05	2.46E − 05	3.28E − 04	8.53E − 06	2.83E − 02	3.17E − 02	1.09E − 01
Summer	TWW	5.08 + 00	2.07 E − 02	4.00E − 02	1.26E − 02	1.07E − 04	1.21E − 03	1.53E − 03	5.62E − 02	2.29E − 03	2.63E − 01	2.40E − 03	2.77E − 03	1.59E − 03	5.16E − 04	2.72E − 05	4.59E − 04	1.52E − 01	8.60E − 03	3.32E − 01
	URW	5.19E + 01	8.90 E − 03	8.62E − 02	2.52E − 02	5.47E − 04	3.11E− 04	4.75E-03	5.59E − 02	7.33E − 01	5.00E − 02	1.16E − 03	3.33E − 04	1.42E − 04	1.56E − 03	1.44E − 03	3.19E − 04	1.08E − 01	1.23E − 02	8.49E − 01
	DRW	2.40E + 02	3.89 E − 02	1.64E − 01	5.43E − 02	3.44E − 04	9.39E− 04	2.56E − 03	2.09E − 01	1.02E + 00	4.94E − 01	6.09E − 03	4.96E − 03	4.80E − 04	6.42E − 04	7.74E − 05	1.36E − 03	6.75E − 01	3.89E − 02	1.74E+ 00

Table S4. Average gene concentrations in wastewater and river water samples (kopii/16S rRNA).

Season	Type of Samples	bfr	HF183 / BacR 287	uidA	*Faecalis* 1	cfxA	bla_AMP-C	tet (Q)	tet(X)	ermF	linA	mefA	catA1	fexA	vanA	bexA	intI1	intI2	ARGs	Cells
	HWW	2.97E − 02	3.47E − 03	3.90E − 04	1.37E − 04	1.24E − 02	4.67E − 05	3.79E − 02	3.92E − 03	3.14E +00	1.88E − 03	3.20E − 03	4.60E − 04	3.59E − 07	5.40E − 04	2.52E − 04	6.84E − 02	4.89E − 04	3.20E+ 00	2.64E − 03
	UWW	5.38E − 04	5.24E − 05	7.04E − 04	9.92E − 05	3.33E − 05	3.73E − 05	2.18E − 03	4.86E − 04	4.06E − 02	2.01E − 05	1.14E − 03	9.05E − 05	1.72E − 06	4.07E − 06	2.48E − 06	4.84E − 02	6.41E − 03	4.46E − 02	2.96E − 03
Winter	TWW	1.64E − 05	2.31E − 04	1.14E − 04	3.15E − 06	1.50E − 07	8.28E − 06	1.25E − 04	9.10E − 07	7.49E − 05	6.10E − 07	9.76E − 07	2.88E − 07	1.19E − 06	2.43E − 07	1.16E − 06	1.41E − 03	1.37E − 04	2.14E − 04	2.11E − 03
	URW	1.99E − 06	4.88E − 06	4.01E − 05	2.29E − 07	3.36E − 08	8.97E − 07	4.66E − 06	5.34E − 07	1.95E − 06	4.67E − 08	7.35E − 08	5.72E − 08	4.92E − 07	5.64E − 08	3.32E − 08	1.45E − 04	8.53E − 06	8.84E − 06	9.96E − 04
	DRW	1.55E − 04	1.92E − 04	2.77E − 05	1.69E − 06	1.51E − 06	1.63E − 06	2.38E − 04	1.29E − 06	1.88E − 04	7.17E − 06	1.40E − 05	4.33E − 07	1.39E − 08	1.58E − 08	3.40E − 06	5.65E − 04	2.99E − 05	4.56E − 04	3.17E − 04

Summer	HWW	4.16E − 02	2.35E − 02	9.08E − 04	3.48E − 05	1.21E − 02	7.64E − 05	3.94E − 02	1.19E − 02	2.77E + 00	2.91E − 03	2.35E − 03	2.66E − 04	5.63E − 07	9.42E − 05	5.90E − 04	2.33E − 02	2.76E − 04	2.84E + 00	2.22E − 03
	UWW	2.23E − 04	7.23E − 05	1.02E − 02	9.73E − 04	3.41E − 05	4.93E − 04	9.41E − 04	8.68E − 03	3.55E − 03	9.89E − 05	1.06E − 02	1.08E − 05	5.55E − 06	7.38E − 05	1.92E − 06	6.37E − 03	7.15E − 03	2.45E − 02	2.25E − 01
	TWW	4.06E − 03	7.88E − 03	2.47E − 03	2.11E − 05	2.38E − 04	3.01E − 04	1.10E − 02	4.51E − 04	5.18E − 02	4.72E − 04	5.45E − 04	3.13E − 04	1.02E − 04	5.35E − 06	9.04E − 05	2.98E − 02	1.69E − 03	6.54E − 02	1.97E − 01
	URW	1.72E − 04	1.66E − 03	4.85E − 04	1.05E − 05	5.98E − 06	9.16E − 05	1.08E − 03	1.41E − 02	9.63E − 04	2.23E − 05	6.42E − 06	2.73E − 06	3.00E − 05	2.77E − 05	6.14E − 06	2.08E − 03	2.37E − 04	1.64E − 02	1.93E − 02
	DRW	1.62E − 04	6.85E − 04	2.27E − 04	1.44E − 06	3.92E − 06	1.07E − 05	8.73E − 04	4.24E − 03	2.06E − 03	2.54E − 05	2.07E − 05	2.00E − 06	2.68E − 06	3.23E − 07	5.67E − 06	2.82E − 03	1.62E − 04	7.25E − 03	4.17E − 03

Table S5. Differences in gene concentrations in the analyzed samples between seasons (Kruskal-Wallis ANOVA; significant results are marked in red, $p < 0.05$).

**Genes**	**Cells**	**16S rRNA**	***bfr***	**HF183/BacR287**	***uid*A**	***Faecalis*1**	***cfx*A**	***bla*$_{AMP-C}$**	***tet*(Q)**	***tet*(X)**	***erm*F**	***lin*A**	***mef*A**	***cat*A1**	***fex*A**	***van*A**	***bex*A**	***int*I1**	***int*I2**	**ARGs**
Bez podziału na sezon	0.0001	0.0005	0.0001	0.0001	0.0001	0.0002	0.0000	0.0002	0.0000	0.0001	0.0001	0.0000	0.0000	0.0001	0.0001	0.0001	0.0001	0.0000	0.0000	0.0000
Winter	0.0157	0.0091	0.0091	0.0090	0.0111	0.0091	0.0083	0.0091	0.0091	0.0144	0.0091	0.0091	0.0091	0.0091	0.0118	0.0117	0.0091	0.0089	0.0091	0.0091
Summer	0.0118	0.0090	0.0091	0.0117	0.0106	0.0262	0.0091	0.0111	0.0091	0.0091	0.0091	0.0091	0.0091	0.0091	0.0091	0.0118	0.0110	0.0121	0.0121	0.0091

Table S6. Correlations between gene concentrations (Spearman's rank correlation coefficient; significant results are marked in red, $p < 0.05$).

**Genes**	**Cells**	**16S rRNA**	***bfr***	**HF183/BacR287**	***uid*A**	***Faecalis*1**	***cfx*A**	***bla*$_{AMP-C}$**	***tet*(Q)**	***tet*(X)**	***erm*F**	***lin*A**	***mef*A**	***cat*A1**	***fex*A**	***van*A**	***bex*A**	***int*I1**	***int*I2**	**ARGs**
Cells		0.70	0.71	0.52	0.86	0.76	0.78	0.83	0.79	0.63	0.79	0.77	0.85	0.85	0.74	0.61	0.72	0.82	0.79	0.72
16S rRNA	0.70		0.72	0.63	0.84	0.84	0.69	0.70	0.74	0.58	0.65	0.70	0.77	0.69	0.50	0.57	0.72	0.86	0.84	0.74
bfr	0.71	0.72		0.91	0.85	0.83	0.96	0.89	0.97	0.80	0.95	0.97	0.94	0.92	0.68	0.67	0.98	0.86	0.87	0.96
HF183/BacR287	0.52	0.63	0.91		0.79	0.70	0.83	0.75	0.86	0.67	0.83	0.85	0.83	0.80	0.55	0.52	0.93	0.78	0.78	0.84
*uid*A	0.86	0.84	0.85	0.79		0.90	0.85	0.88	0.90	0.70	0.84	0.88	0.93	0.89	0.67	0.63	0.89	0.95	0.94	0.85
*Faecalis*1	0.76	0.84	0.83	0.70	0.90		0.82	0.89	0.86	0.67	0.79	0.83	0.89	0.85	0.68	0.72	0.83	0.93	0.93	0.82
*cfx*A	0.78	0.69	0.96	0.83	0.85	0.82		0.89	0.98	0.85	0.98	0.98	0.94	0.94	0.76	0.75	0.94	0.85	0.87	0.97
bla$_{AMP-C}$	0.83	0.70	0.89	0.75	0.88	0.89	0.89		0.88	0.79	0.90	0.91	0.93	0.93	0.82	0.76	0.88	0.86	0.88	0.86
tet(Q)	0.79	0.74	0.97	0.86	0.90	0.86	0.98	0.88		0.80	0.97	0.97	0.95	0.94	0.71	0.71	0.96	0.92	0.91	0.97
tet(X)	0.63	0.58	0.80	0.67	0.70	0.67	0.85	0.79	0.80		0.86	0.86	0.79	0.83	0.86	0.80	0.76	0.68	0.71	0.87
*erm*F	0.79	0.65	0.95	0.83	0.84	0.79	0.98	0.90	0.97	0.86		0.97	0.93	0.95	0.80	0.76	0.93	0.84	0.85	0.96
*lin*A	0.77	0.70	0.97	0.85	0.88	0.83	0.98	0.91	0.97	0.86	0.97		0.93	0.93	0.74	0.72	0.96	0.85	0.87	0.97
*mef*A	0.85	0.77	0.94	0.83	0.93	0.89	0.94	0.93	0.95	0.79	0.93	0.93		0.97	0.76	0.69	0.93	0.93	0.94	0.93
*cat*A1	0.85	0.69	0.92	0.80	0.89	0.85	0.94	0.93	0.94	0.83	0.95	0.93	0.97		0.83	0.71	0.90	0.90	0.90	0.91

*fex*A	0.74	0.50	0.68	0.55	0.67	0.68	0.76	0.82	0.71	0.86	0.80	0.74	0.76	0.83		0.86	0.65	0.67	0.69	0.71
*van*A	0.61	0.57	0.67	0.52	0.63	0.72	0.75	0.76	0.71	0.80	0.76	0.72	0.69	0.71	0.86		0.63	0.63	0.71	0.73
*bex*A	0.72	0.72	0.98	0.93	0.89	0.83	0.94	0.88	0.96	0.76	0.93	0.96	0.93	0.90	0.65	0.63		0.88	0.88	0.94
*int*I1	0.82	0.86	0.86	0.78	0.95	0.93	0.85	0.86	0.92	0.68	0.84	0.85	0.93	0.90	0.67	0.63	0.88		0.96	0.86
*int*I2	0.79	0.84	0.87	0.78	0.94	0.93	0.87	0.88	0.91	0.71	0.85	0.87	0.94	0.90	0.69	0.71	0.88	0.96		0.88
ARGs	0.72	0.74	0.96	0.84	0.85	0.82	0.97	0.86	0.97	0.87	0.96	0.97	0.93	0.91	0.71	0.73	0.94	0.86	0.88	

Table S7. A comparison of gene concentrations in URW and DRW (Mann-Whitney U test; significant results are marked in red, $p < 0.05$).

Variable	Sum of URW ranks	Sum of DRW U ranks	U	Z	*p*	Z (with the continuity correction)	*p*	N	N	2 * 1str.
Cells	35.0	43.0	14.0	-0.56	0.58	−0.56	0.58	6	6	0.59
16S rRNA	30.0	48.0	9.0	−1.36	0.17	−1.36	0.17	6	6	0.18
bfr	21.0	57.0	0.0	−2.80	0.01	−2.80	0.01	6	6	0.00
HF183/BacR287	21.0	57.0	0.0	−2.80	0.01	−2.81	0.00	6	6	0.00
*uid*A	22.0	56.0	1.0	−2.64	0.01	−2.64	0.01	6	6	0.00
*Faecalis*1	30.0	48.0	9.0	−1.36	0.17	−1.36	0.17	6	6	0.18
*cfx*A	21.0	57.0	0.0	−2.80	0.01	−2.82	0.00	6	6	0.00
bla_{AMP-C}	30.0	48.0	9.0	−1.36	0.17	−1.36	0.17	6	6	0.18
tet(Q)	21.0	57.0	0.0	−2.80	0.01	−2.80	0.01	6	6	0.00
tet(X)	30.0	48.0	9.0	−1.36	0.17	−1.36	0.17	6	6	0.18
*erm*F	21.0	57.0	0.0	−2.80	0.01	−2.80	0.01	6	6	0.00
*lin*A	21.0	57.0	0.0	−2.80	0.01	−2.80	0.01	6	6	0.00
*mef*A	21.0	57.0	0.0	−2.80	0.01	−2.80	0.01	6	6	0.00
*cat*A1	21.0	57.0	0.0	−2.80	0.01	−2.80	0.01	6	6	0.00
*fex*A	49.0	29.0	8.0	1.52	0.13	1.52	0.13	6	6	0.13
*van*A	48.0	30.0	9.0	1.36	0.17	1.36	0.17	6	6	0.18
*bex*A	21.0	57.0	0.0	−2.80	0.01	−2.80	0.01	6	6	0.00
*int*I1	21.0	57.0	0.0	−2.80	0.01	−2.81	0.00	6	6	0.00
*int*I2	21.0	57.0	0.0	−2.80	0.01	−2.80	0.01	6	6	0.00
ARGs	21.0	57.0	0.0	−2.80	0.01	−2.80	0.01	6	6	0.00
